# Heat stress inhibits TLR4-NF-κB and TLR4-TBK1 signaling pathways in broilers infected with Salmonella Typhimurium

**DOI:** 10.1007/s00484-021-02146-5

**Published:** 2021-06-01

**Authors:** Wei-Hao Li, Yi-Lei Liu, Jian-Chi Lun, Yong-Ming He, Lu-Ping Tang

**Affiliations:** School of Life Science and Engineering, Foshan University, Foshan, 528225 China

**Keywords:** Broiler, Caspase-1, Intestinal immunity, Inflammation, NLRP3

## Abstract

With the global warming, the harm of heat stress (HS) to the breeding industry has become more common, which causes the decline of animal production performance and low immunity. This study aimed to analyze the effect of HS on the intestinal immune function of *Salmonella-*infected chickens. Fourteen-day-old broilers were divided into the following four groups of eight replicates: control (Control), heat stress (HS), Salmonella Typhimurium (ST), and heat stress + Salmonella Typhimurium (HS+ST). The broilers were subjected to a heat stress of 35 °C from 15 to 28 days of age. Salmonella Typhimurium (ST, 14028, 10^9^ cfu/mL) was inoculated, via oral administration at 29 days of age, into ST and HS+ST group birds. On the 4th day after Salmonella Typhimurium administration, an increase in jejunum IgA levels was observed in chickens infected with Salmonella Typhimurium. Mechanistic regulation of TLR4-NFκB-NLRP3 and TLR4-TBK1 signaling by heat stress was evaluated in Salmonella Typhimurium–infected broilers. Heat stress markedly inhibited the expression of cytokines including TNF-α, IL-6, IL-1β, NLRP3, caspase-1, NF-κB-p65, and p-NF-κB-p65, and the TLR4-TBK1 cytokines IFN-α, IFN-γ, p-IRF3, and p-TBK1 in jejunum of broilers infected with Salmonella Typhimurium. Collectively, our results demonstrate that heat stress can inhibit intestinal immune response by downregulating the expression of TLR4-NFκB-NLRP3 and TLR4-TBK1 signaling pathways in broilers infected with Salmonella Typhimurium.

## Introduction

Heat stress (HS) is a reality in the poultry industry in tropical countries for a long time. Other factors in the animal facilities, or animal density and others, could also induce increase of temperature of the production system can increase or cause HS in poultry production. The global warming will boost the issue. Broilers lack sweat glands on the skin, and genetic selection also decreases their heat tolerance to enable rapid growth rates, thereby rendering broilers particularly susceptible to high ambient temperatures (Varasteh et al., 2015). Extensive research has shown that heat stress affects broiler physiology, resulting in the occurrence of various physiological disorders such as slow growth and accelerated death rate (Quinteiro-Filho et al., 2012). Additionally, it has been previously observed that HS disrupts intestinal integrity in response to injury (Garriga et al., 2006), particularly affecting the integrity of the intestinal epithelium and triggering immunosuppression (Abdelqader et al., 2017; Pearce et al., 2013) and inflammation (Burkholder et al., 2008).

Studies show that environmental stress may induce colonization and shedding of intestinal pathogens and promote the transmission of pathogens between animals (Rychlik & Barrow, 2005). Furthermore, acute heat stress can transform the epithelial structure of the normal intestine (Alhenaky et al., 2017), which may lead to increased attachment and colonization of *Salmonella Enteritidis* (Sellin et al., 2014). In young chickens infected with *Salmonella*, decreased growth performance and intestinal lesions, as well as inflammation and injury of intestinal permeability, have been observed (Alhenaky et al., 2017). Salmonella Typhimurium, a gram-negative intracellular pathogen, is responsible for 1.3 billion cases of gastroenteritis and 3 million deaths worldwide each year (Dunkley et al., 2009). Salmonella Typhimurium colonizes the small intestine and causes enterotoxins to disrupt the intestinal barrier, triggering inflammation of the intestinal mucosa, and then causes bacteremia which results in the development of severe intestinal inflammation (Broz et al., 2012; Wotzka et al., 2017). A combinatorial effect of HS and *Salmonella Enteritidis* infection has been reported to damage the intestinal barrier, eventually leading to the development of inflammatory infiltrates in the intestine (Quinteiro-Filho et al., 2012). However, the studies on intestinal immunity in chickens treated with heat stress and pathogenic bacteria are limited.

Upon bacterial entry, it is specifically recognized by TLR4 and induces various proinflammatory cytokines, including TNF-α, IL-8, IL-6, and IL-1β. The expression of pro-inflammatory cytokines is regulated by NF-κB expression. Type I interferon can be induced by TBK1, which inhibits pathogens such as *Salmonella*, *enteropathogenic E. coli* to thrive in the host. This study specifically aimed to investigate the effects of heat stress on the immunological response of Salmonella Typhimurium–infected broilers. We will study the protein levels of TLR4, NF-κB, NLRP3, Caspase-1, TNF-α, IL-6, IL-1β, TBK1, IRF3, IFN-α, and IFN-γ in jejunum of broilers to measure the changes of intestinal immune function.

## Materials and methods

### Broilers

A total of 32 one-day-old male broilers were purchased from a commercial hatchery (Foshan Nanhai Poultry Corporation, Foshan, China). One-day-old broilers were housed in climate-controlled rooms at Foshan University (Guangdong, China). Throughout the experiment, broilers were maintained in isolator chambers with high-efficiency particulate air (HEPA) filters. The broilers were provided with water and feed ad libitum. The humidity was monitored and maintained at 60%.

### Ethical considerations

Care and handling of chickens were performed in compliance with the Animal Ethics Committee Guidelines of the Academy of National Food and Strategic Reserves Administration (Beijing, China). Permission to use samples was also granted by the Ethical Clearance Committee of Foshan University.

### Group and heat stress

From days 1 to 14 of life (ED1 to ED14), the broilers were maintained at the recommended environmental temperatures (33 °C ± 1 °C from ED1 to ED7 and 28 °C ± 1 °C from ED7 to ED14). At ED15, the broilers were randomly divided into four groups with eight birds in each group.

The treatments were as follows: control group (Control), heat stress group (HS), Salmonella Typhimurium group (ST), and heat stress + Salmonella Typhimurium (HS + ST) group. Broilers in the HS + ST group and HS group were subjected to 35 °C ± 2 °C heat stress for 2 weeks (14–28 days of age); then, the broilers in the ST and HS+ST groups were inoculated with 1-mL suspension of Salmonella Typhimurium (1×10^9^ cfu/mL) by oral administration, and broilers in the control and HS groups were treated with sterile saline only. On the 4th day after Salmonella Typhimurium challenge (ED32), the broilers were euthanized by CO_2_ inhalation and jejunum samples were collected immediately for histological observation and examination.

### Inoculation of the Salmonella Typhimurium strain

The Salmonella Typhimurium strain was originally obtained from the Guangdong Microbial Culture Collection Center (Guangdong, China). The frozen strain was thawed and incubated with 1×10^9^ CFU/mL of the inoculum. On day 29, 1 mL of the inoculum (10^9^ cfu/mL of *Salmonella Typhimurium*) was orally administered into birds in the ST and the HS + ST group, using a lavage apparatus.

### Histochemistry of the jejunum

The jejunum tissues were fixed in 10% buffered formalin, embedded in paraffin, and cut into 5-μm-thick sections. The sections were stained with hematoxylin and eosin (H&E) for analysis by light microscopy (Nikon ECLIPSE E200, Tokyo, Japan).

### Western blot analysis

Briefly, the jejunum tissues were homogenized using a protein lysis buffer, separated on a 10% SDS-polyacrylamide gel, and electroblotted onto PVDF membranes. Membrane blots were incubated with antibodies (anti-Hsp70, anti-TLR4, anti-TBK1, anti-p-TBK1, anti-IRF3, anti-p-IRF3, anti-IFN-α, anti-IFN-γ, anti-NLRP3, anti-Caspase-1, anti-pro-IL-1β, anti-IL-1β, anti-NF-κB p65, anti-p-NF-κB p65, anti-IκB α, anti-p-IκB α, anti-IL-6, anti-TNF-α, anti-HMGB1, and anti-β-actin) (Cell Signaling Technology, Boston, USA) for 2 h, at room temperature. The membranes were subjected to washing steps using TBST buffer thrice, followed by 1 h of incubation with HRP-conjugated secondary anti-mouse (or anti-rabbit) IgG from goat. Protein expression was analyzed using the ImageJ software.

### Intestinal IgA determination

The birds were euthanized by CO_2_ inhalation and jejunums were collected. Jejunums were cleaned with PBS, and 0.1-g jejunum samples were removed in 0.9 mL PBS and homogenized. The mixture was centrifuged 3000*g* for 20 min at 4 °C. Then, the supernatant was collected for further test. Total IgA jejunum levels were detected using an enzyme-linked immunosorbent assay (ELISA) kit purchased from Jiangsu Meimian industrial Co., Ltd. in accordance with the manufacturer’s protocol.

### Statistical analyzes

The data are presented as the mean ± standard deviation. Statistical significance among treatment groups was calculated using one-way and two-way ANOVAs (SPSS 17.0). Different letters indicate significant differences between treatments (P *<*0.05). ^*^P *<*0.05 indicates the interaction between heat stress and *Salmonella* infection.

## Results

### Heat stress induces changes in the intestinal morphology in ST-infected broilers

**Fig. 1:**
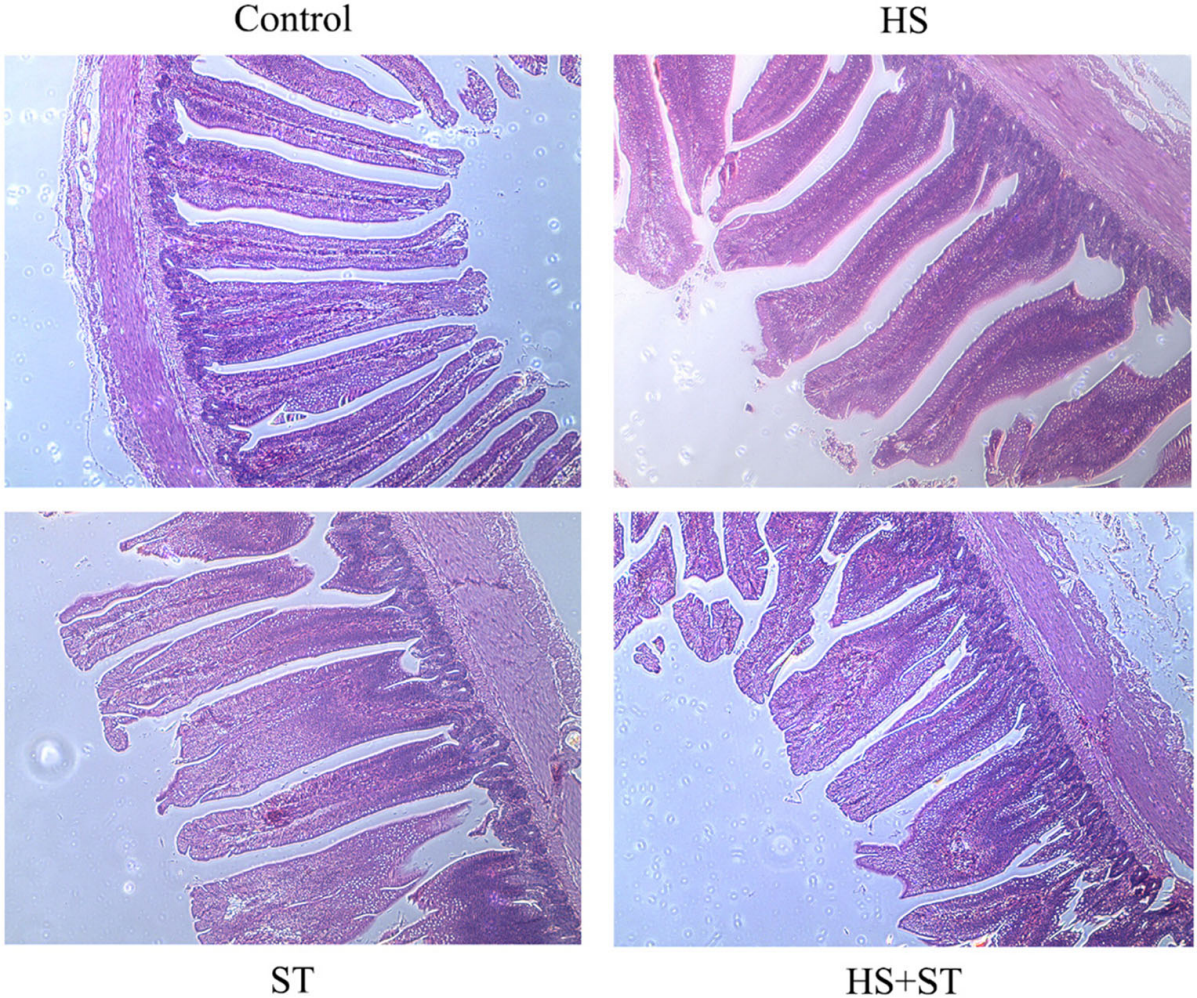
Effects of heat stress on jejunum morphology in ST-infected broilers (H&E staining, magnification: ×100)

The villi in the jejunum showed normal arrangement and displayed a regular shape in the control broilers (Fig. 1), while the microvilli were markedly damaged in the HS broilers. Additionally, the villi of the ST group broilers were missing or shortened, and the mucosal villi were edematous and enlarged. The width of lamina propria and epithelial was increased, and there was little inflammatory infiltration in ST group broilers, compared with control broilers. The injury showed worse outcomes in the HS+ST group after subjection to heat stress, expressed more serious inflammatory infiltration, villi shortened, and the mucosal villi edematous.

### Heat stress increases the jejunum IgA levels in ST-infected broilers

**Fig. 2:**
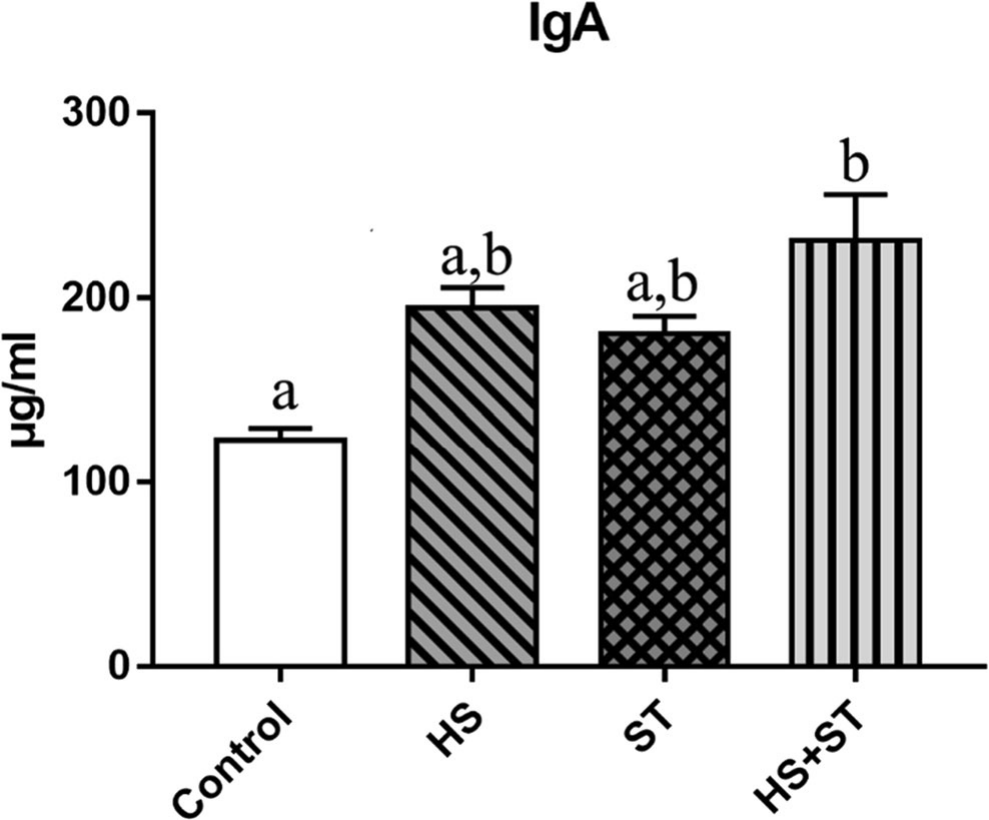
Effects of heat stress on the jejunum IgA levels in ST-infected broilers. Extracted intestinal mucus samples were titrated for IgA level determination. Different letters indicate significant differences between treatments (*P <*0.05). ^*^P <0.05 indicates the interaction between heat stress and *Salmonella* infection. n = 8/group

As shown in Fig. 2, heat stress and ST administration significantly increased the jejunum IgA levels (*P*<0.05 and *P*<0.05, respectively). IgA levels were significantly higher in the HS+ST group than those in the ST group (*P*<0.05).

### Heat stress decreases TBK1-activated interferons in the jejunum of ST-infected broilers.

**Fig. 3:**
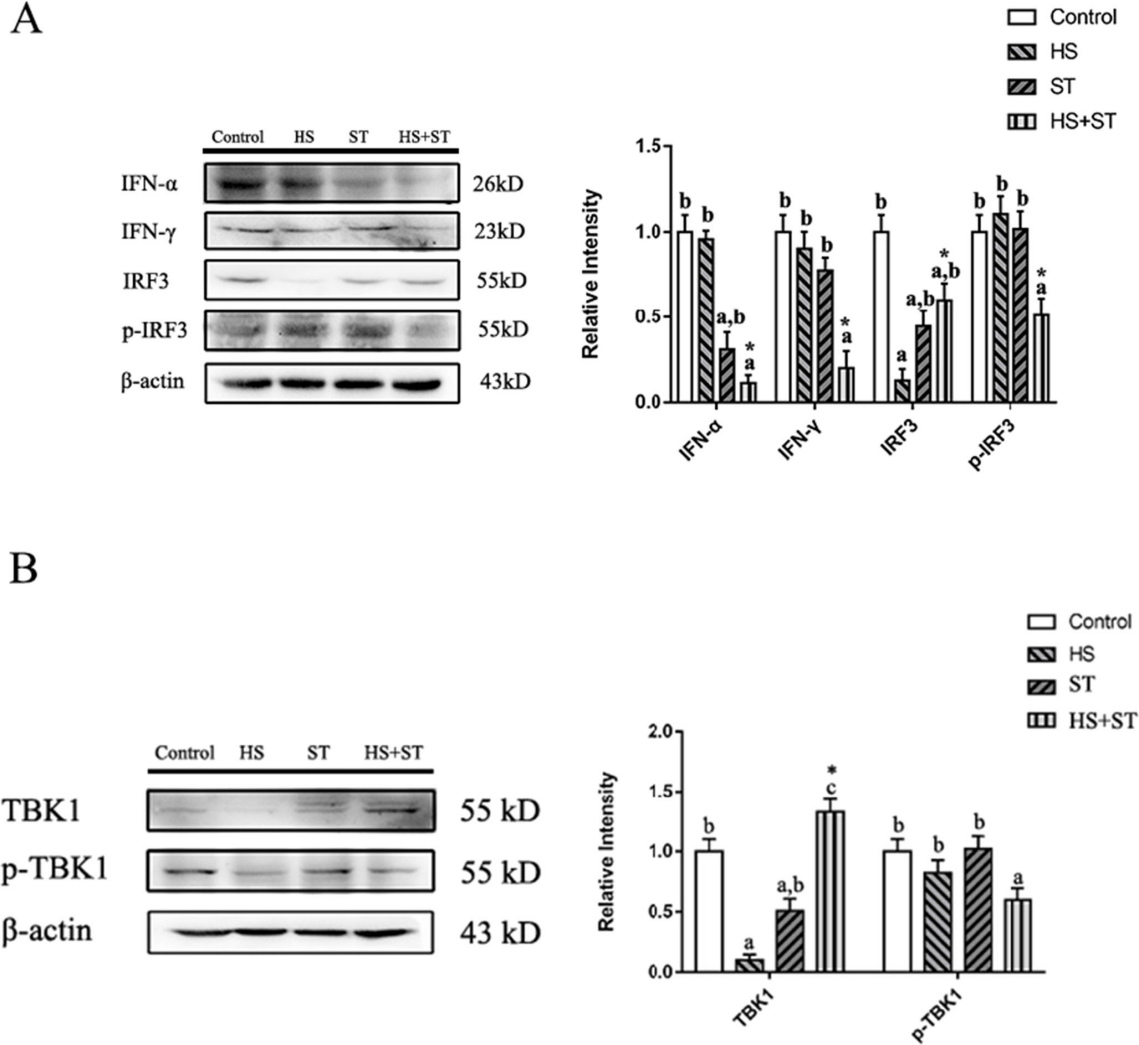
Effects of heat stress on the jejunum TBK1-IRF3 and interferon protein expression levels in ST-infected broilers. (A) IRF3, p-IRF3, and interferon protein expression levels and (B) TBK1 and p-TBK1 protein expression levels in the jejunum of broilers were determined by performing western blot analysis. Different letters indicate significant differences between treatments (*P <*0.05). ^*^P <0.05 indicates the interaction between heat stress and *Salmonella* infection

As shown in Fig. 3A, compared with control group, ST infection decreased the jejunum protein expression of IFN-α (*P<*0.05). However, a significantly lower protein level of IFN-α and IFN-γ was observed (*P<*0.05 and *P<*0.05) in the HS+ST group than that in the ST group. HS and ST infection alone decreased the protein expression of IRF3 (*P<*0.05 and *P<*0.05, respectively) in the jejunum of broilers compared with control group broilers. There was no significant difference in p-IRF3 expression levels between the HS and control groups. However, heat-stressed broilers infected with ST showed reduced protein expression of p-IRF3 compared with the ST group (*P<*0.05). There had been an interaction between heat stress and *Salmonella* infection in the expressions of IFN-α, IFN-γ, IRF3, and p-IRF3.

As shown in Fig. 3B, the individually administered HS and ST infections decreased the protein expression of TBK1 in the jejunum of broilers (*P<*0.05 and *P<*0.05, respectively). There was no significant difference in p-TBK1 levels between the heat stress and ST group birds. However, in the HS+ ST group, the protein expression of TBK1 was increased (*P<*0.05) and p-TBK1 was significantly reduced (*P<*0.05) compared with the ST group.

### Heat stress inhibits NLRP3/Caspase-1 and inflammatory cytokines protein expressions in the jejunum of ST-infected broilers

**Fig. 4:**
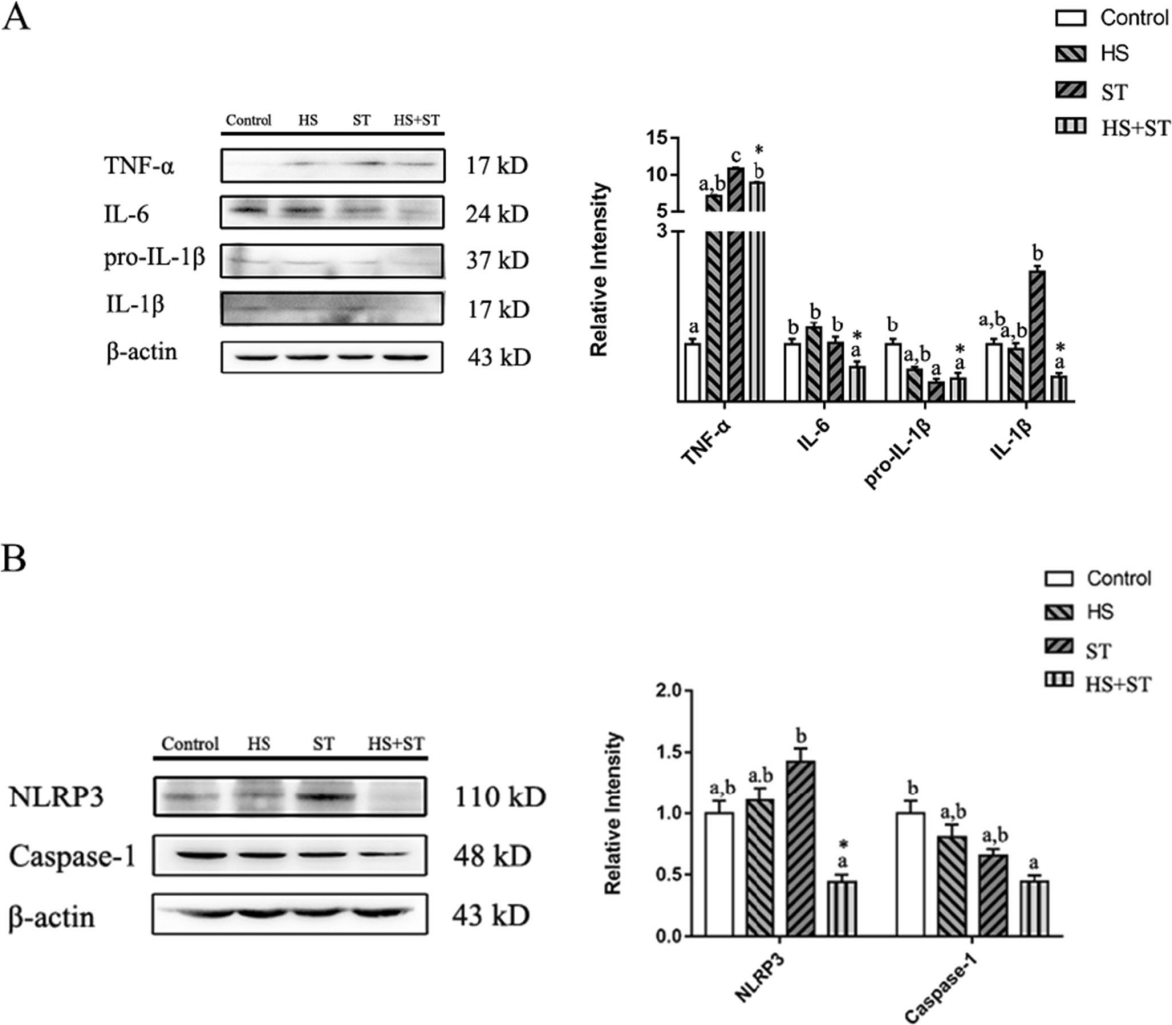
Effects of heat stress on NLRP3 and caspase-1 protein expression levels in ST-infected broilers. (A) TNF-α, IL-6, pro-IL-1β, and IL-1β protein expression levels and (B) NLRP3 and Caspase-1 protein expression levels in the jejunum of broilers were determined by performing western blot analysis. Different letters indicate significant differences between treatments (*P <*0.05). ^*^P <0.05 indicates the interaction between heat stress and *Salmonella* infection

As shown in Fig. 4A, broilers in the ST group exhibited higher TNF-α and IL-1β levels and lower pro-IL-1β protein levels than those in the control group (*P* < 0.05, *P* < 0.05, and *P* < 0.05, respectively). Broilers in the HS+ST group exhibited lower TNF-α, IL-6, and IL-1β protein levels than those in the ST group (*P* < 0.05, *P* < 0.05, and *P* < 0.05, respectively).

As shown in Fig. 4B, broilers infected with ST showed increased NLRP3 expression levels but decreased Caspase-1 protein expression levels compared to those in the control group (*P* < 0.05 and *P* < 0.05). Broilers in the HS+ST group exhibited lower NLRP3 and Caspase-1 protein levels than those in the ST group (*P* < 0.05 and *P* < 0.05, respectively). There had been an interaction between heat stress and *Salmonella* infection in the expressions of TNF-α, IL-6, IL-1β, pro-IL-1β, and NLRP3.

### Heat stress decreases the phosphorylation of NF-κB p65 and IκB-α in the jejunum of broilers infected with ST

**Fig. 5:**
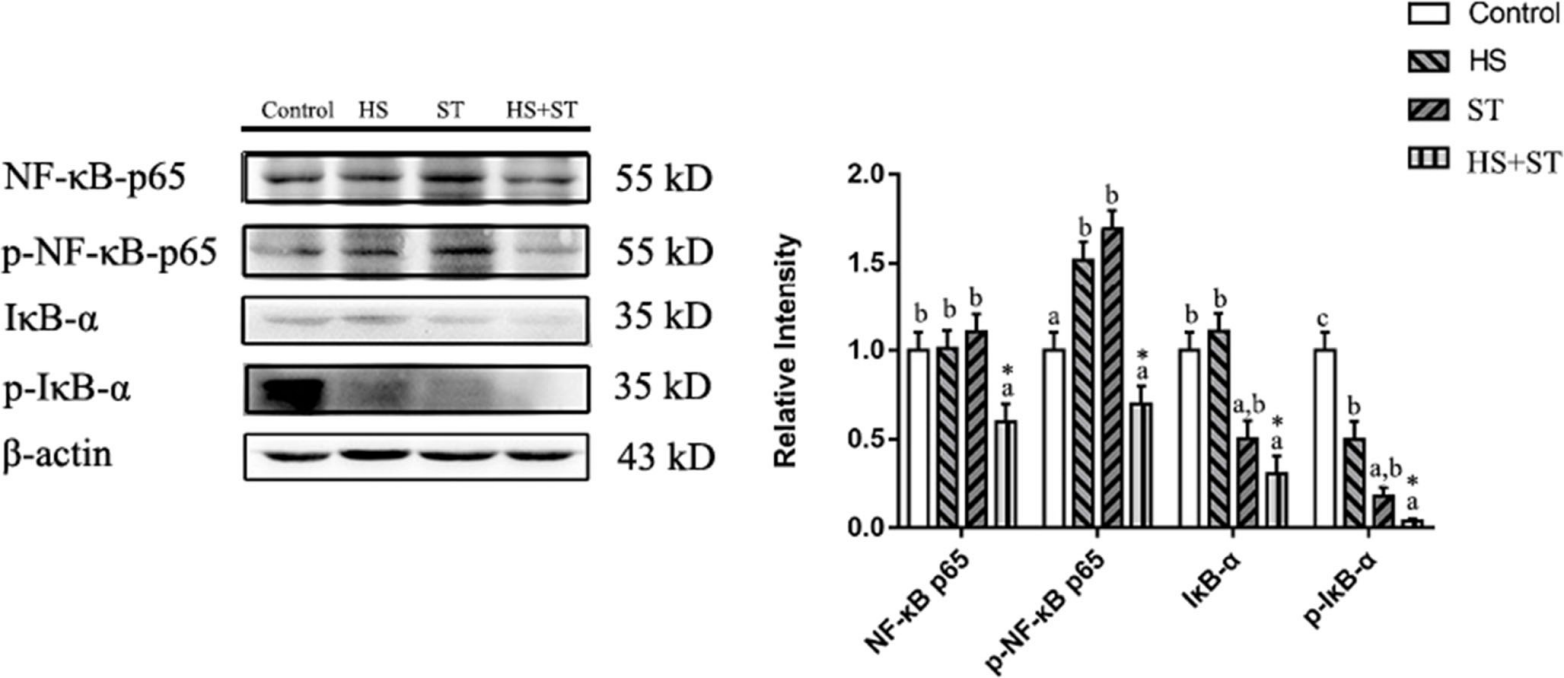
Effects of heat stress on the NF-κB signaling pathway expression levels in ST-infected broilers. Protein expression levels in the jejunum of broilers were determined by performing western blot analysis. Different letters indicate significant differences between treatments (*P <*0.05). ^*^P <0.05 indicates the interaction between heat stress and *Salmonella* infection

As shown in Fig. 5, broilers in the ST group exhibited higher p-NF-κB p65 and lower IκB-α and p-IκB-α protein levels than those in the control group (*P <* 0.05, *P <* 0.05, and *P <* 0.05, respectively). HS also increased p-NF-κB p65 levels and decreased p-IκB-α protein levels compared with those in the control group (*P <* 0.05 and *P <* 0.05, respectively). Broilers in the HS+ST group exhibited lower NF-κB p65, p-NF-κB p65, and p-IκB-α protein levels than those in the ST group (*P <* 0.05, *P <* 0.05, and *P <* 0.05, respectively).

### Heat stress decreases protein expression of TLR4 and HSP70 in the jejunum of broilers infected with ST

**Fig. 6:**
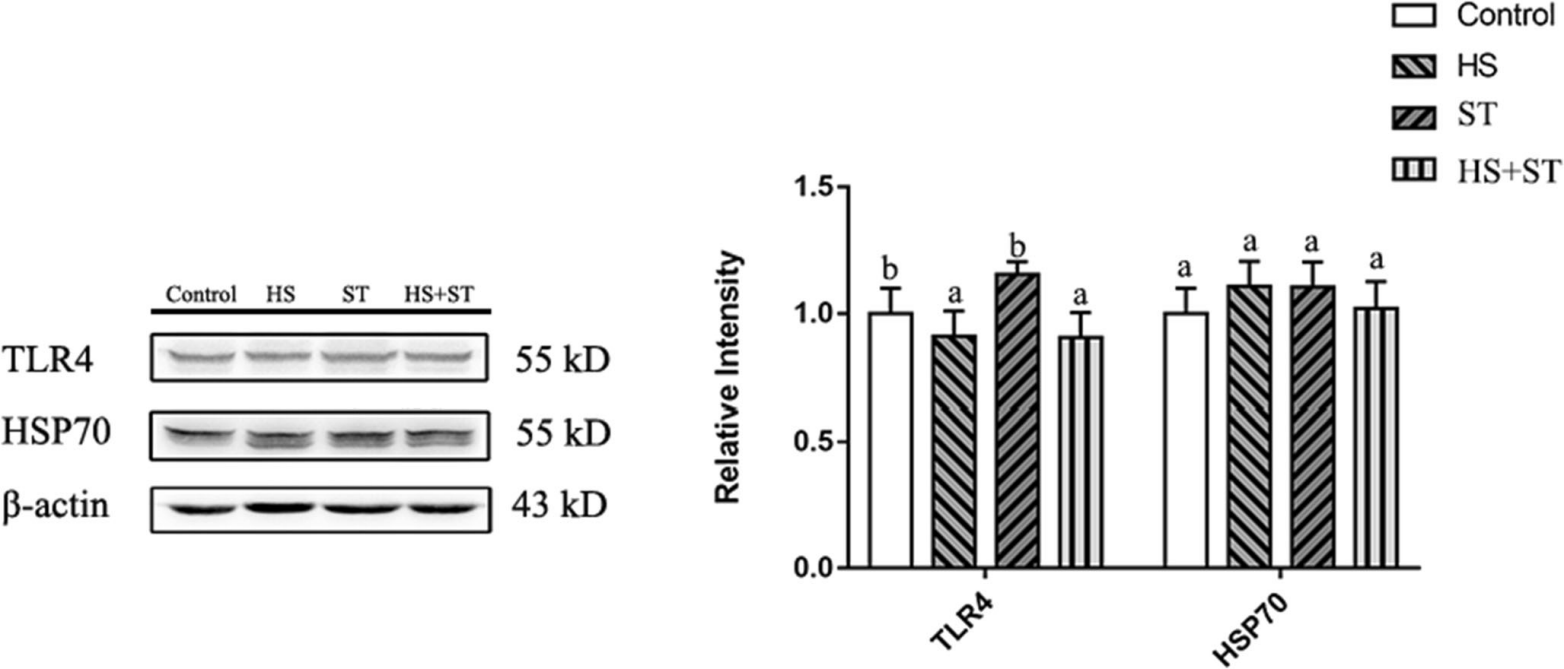
Effects of heat stress on TLR4 and Hsp70 protein expression levels in ST-infected broilers. Protein expression levels in the jejunum of broilers were determined by performing western blot analysis. Different letters indicate significant differences between treatments (*P <*0.05). ^*^P <0.05 indicates the interaction between heat stress and *Salmonella* infection

As shown in Fig. 6, HS decreased TLR4 protein expression in the HS+ST group (*P < 0*.05) compared with the ST group. There were no significant differences in HSP70 protein levels among the groups (*P >* 0.05).

## Discussion

Immunosuppression is a major challenge in chronic stress, especially when the animals are challenged with *Salmonella* (Calefi et al., 2016; Quinteiro-Filho et al., 2017; Alhenaky et al., 2017). Heat stress can restrain intestinal barrier function, which may be attributed to the suppression of the mucosal immune system, particularly in animals with intestinal infections (Mestecky, 1987). IgA plays a crucial role against bacterial pathogens and viral and parasitic infections. Mice infected with Salmonella Typhimurium showed increased IgA levels in the intestinal lamina propria (Allen et al., 2000). HS or Salmonella Typhimurium infection alone significantly enhanced the IgA levels, and HS chickens infected with Salmonella Typhimurium showed higher IgA levels than those infected with Salmonella Typhimurium alone. These results suggest that heat stress increases IgA levels, which may result in the elicitation of a strong local immune response to Salmonella Typhimurium invasion.

Upon bacterial entry, Toll like receptor 4 (TLR4) endotoxin lipopolysaccharide (LPS) is specifically recognized by TLR4 and induces macrophages to express various proinflammatory cytokines, including TNF-α, IL-8, IL-6, and IL-1β (Ramachandran, 2014). IL-1β deficiency increases both severity and mortality rate associated with *Salmonella* infection (Eckmann & Kagnoff, 2001). Evidence suggests that IL-6 secretion is a crucial factor in broiler chickens that confers protection against *Salmonella* infection (Chappell et al., 2009). In this study, HS significantly inhibited the protein expression of TNF-α, IL-6, and IL-1β in chickens infected with Salmonella Typhimurium, indicating that reduction in cytokines might result in successful Salmonella Typhimurium invasion, which was consistent with the findings reported by Quinteiro-Filho (Quinteiro-Filho et al., 2017). Innate immunity helps to eliminate pathogens and confers cellular protection through the production of pro-inflammatory cytokines, and the downregulation of these cytokines before resolving inflammation stress may tend to favor *Salmonella* colonization (Malago et al., 2002; Nemeth et al., 2006; Yoo et al., 2000).

The expression of pro-inflammatory cytokines is regulated by nuclear factor kappa-B (NF-κB) expression. TLR4 plays an important role in elicitation of an early host defense against invading pathogens and subsequent occurrence of the inflammatory response (Akira & Takeda, 2004; Newton & Dixit, 2012; Wittebole et al., 2010). Upon LPS stimulation, TLR4 activates the IKK complex to induce the phosphorylation of IκBs, and IκBs translocate into the nucleus to regulate the production of inflammatory cytokines (Akira & Takeda, 2004; Beutler, 2004; Guijarro-Munoz et al., 2014; Newton & Dixit, 2012). Based on our data, the levels of TLR4 were decreased in the heat stress-induced and Salmonella Typhimurium–infected chickens as compared to the Salmonella Typhimurium group birds, which suggested that heat stress might impair early host defense against *Salmonella* invasion. Heat stress also decreased the protein expression levels of p-NF-κB p65 and p-IκBα in chickens infected with Salmonella Typhimurium, and the levels of TLR4 were also decreased, suggesting that heat stress might regulate TLR4-NF-κB signaling in broiler chickens infected with Salmonella Typhimurium.

Caspase-1, as an intracellular inflammasome, is considered a mechanism of intestinal epithelial cells to resist microbial pathogens (Martinon & Tschopp, 2004). Activated caspase-1 induces the maturation of the proinflammatory cytokines IL-1β and IL-18 (Sansonetti et al., 2000), which play pivotal roles in resisting an invasion from a variety of pathogens (Mariathasan et al., 2005). Studies have shown that Casp1 transcript levels decrease slightly with the progression of Salmonella infection, and the lack of caspase-1 renders the body more susceptible to pathogens (Sellin et al., 2014). The results confirmed that heat stress may render chickens more susceptible to *Salmonella* by decreasing capase-1 protein levels in heat-stressed and ST-infected chickens. Research has shown that NLRP3 can activate caspase-1 and cleave pro-IL-1β to IL-1β (Bergsbaken et al., 2009). A significant decrease in NLRP3 expression was observed, indicating that it played a mechanistic role in caspase-1-mediated IL-1β inhibition upon occurrence of heat stress in ST-infected chickens. LPS stimulation triggered NF-κB-mediated upregulated expression of NLRP3 and pro-IL-1β (Bauernfeind et al., 2009). These results suggest that heat stress may regulate TLR4-NF-κB-NLRP3 signaling in broiler chickens infected with ST.

Few studies have reported that type I interferon maintains barrier function by regulating tight junction proteins in intestinal epithelial cells infected by *enteropathogenic E. coli* (Long et al., 2014). In our study, the levels of IFN-γ and IFN-α were decreased in heat-stressed and ST-infected chickens. Following viral infection, a protein named recombinant TANK binding kinase 1 (TBK1) sends a signal that induces a protective program to inhibit viral replication (Yuichiro Tojima et al., 2000). TBK1 deficiency enables pathogens such as *Salmonella*, *enteropathogenic E. coli*, and group A *Streptococci* to thrive easily in the host cytoplasm (Perrin et al., 2004). *Salmonella* has flagellin and LPS, which stimulate TLR4 expression in the lamina propria (Long et al., 2014). TBK1-IKKε mediates the phosphorylation of IRF3 and induces the expression of type I interferon (Doyle et al., 2002; Eva et al., 2004). Finally, p-TBK1 was decreased in the jejunum of heat-stressed broiler chickens infected with ST. Consistent with the heat stress-mediated suppression of TLR4-induced IFN-α expression, the inhibition of p-IRF3 and p-TBK1 expression in heat stressed chickens infected with ST was also observed, suggesting that heat stress inhibited IFN-α activation by inhibiting TBK1 activation.

## Conclusion

**Fig. 7:**
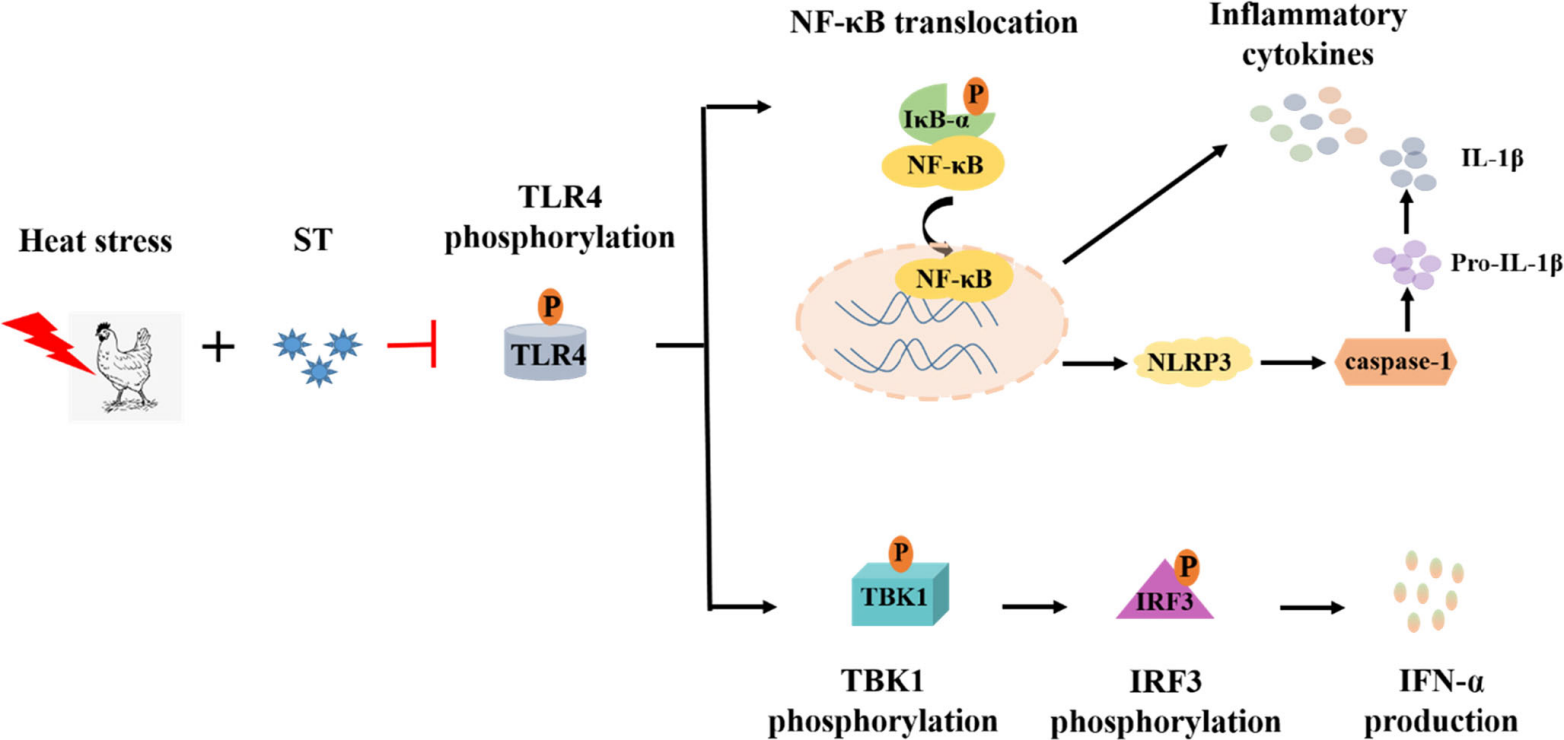
Heat stress inhibits the activation of the TLR4-NF-κB/TBK1 signaling pathway in broilers infected with Salmonella Typhimurium

Our results indicate that heat stress impairs the intestinal immune function in broilers, resulting in increased susceptibility of the broilers to *Salmonella Typhimurium* infection. In summary, our study demonstrates altered TLR4-mediated TBK1 and NF-κB signaling in heat stress-induced broilers infected with *Salmonella* (Fig. 7). These findings may provide a convincing scientific rationale for heat stress-induced lowering of intestinal immunity in poultry.

## References

[CR1] Abdelqader A, Abuajamieh M, Hammad H, Al-Fataftah A (2017). Effects of dietary butyrate supplementation on intestinal integrity of heat-stressed cockerels. J Anim Physiol Anim Nutr.

[CR2] Akira S, Takeda K (2004). Toll-like receptor signalling. Nat Rev Immunol.

[CR3] Alhenaky A, Abdelqader A, Abuajamieh M, Al-Fataftah AR (2017). The effect of heat stress on intestinal integrity and Salmonella invasion in broiler birds. J Therm Biol.

[CR4] Allen JS, Dougan G, Strugnell RA (2000). Kinetics of the mucosal antibody secreting cell response and evidence of specific lymphocyte migration to the lung after oral immunization with attenuated *S. enterica var*. typhimurium. FEMS Immunol Med Microbiol.

[CR5] Bauernfeind FG, Horvath G, Stutz A, Alnemri ES, MacDonald K, Speert D, Fernandes-Alnemri T, Wu J, Monks BG, Fitzgerald KA, Hornung V, Latz E (2009). Cutting edge: NF-kappaB activating pattern recognition and cytokine receptors license NLRP3 inflammasome activation by regulating NLRP3 expression. J Immunol.

[CR6] Bergsbaken T, Fink SL, Cookson BT (2009). Pyroptosis: host cell death and inflammation. Nat Rev Microbiol.

[CR7] Beutler B (2004). Inferences, questions and possibilities in Toll-like receptor signalling. Nature.

[CR8] Broz P, Ohlson MB, Monack DM (2012). Innate immune response to Salmonella typhimurium, a model enteric pathogen. Gut Microbes.

[CR9] Burkholder KM, Thompson KL, Einstein ME, Applegate TJ, Patterson JA (2008). Influence of stressors on normal intestinal microbiota, intestinal morphology, and susceptibility to Salmonella enteritidis colonization in broilers. Poult Sci.

[CR10] Calefi AS, da Silva Fonseca JG, Hamada Cohn DW, Bueno Honda BT, Costola-de-Souza C, Tsugiyama LE, Quinteiro-Filho WM, Piantino Ferreira AJ, Palermo-Neto J (2016). The gut-brain axis interactions during heat stress and avian necrotic enteritis. Poult Sci.

[CR11] Chappell L, Kaiser P, Barrow P, Jones MA, Johnston C, Wigley P (2009). The immunobiology of avian systemic salmonellosis. Vet Immunol Immunopathol.

[CR12] Doyle S, Vaidya S, O’Connell R, Dadgostar H, Dempsey P, Wu T, Rao G, Sun R, Haberland M, Modlin R, Cheng G (2002). IRF3 mediates a TLR3/TLR4-specific antiviral gene program. Immunity.

[CR13] Dunkley KD, Callaway TR, Chalova VI, McReynolds JL, Hume ME, Dunkley CS, Kubena LF, Nisbet DJ, Ricke SC (2009). Foodborne salmonella ecology in the avian gastrointestinal tract. Anaerobe.

[CR14] Eckmann L, Kagnoff MF (2001). Cytokines in host defense against Salmonella. Microbes Infect.

[CR15] Eva M, Pålsson-McDermott, O’Neill LAJ (2004). Signal transduction by the lipopolysaccharide receptor, toll-like receptor-4. Insect Sci.

[CR16] Garriga C, Hunter RR, Amat C, Planas JM, Mitchell MA, Moreto M (2006). Heat stress increases apical glucose transport in the chicken jejunum. Am J Physiol-Regul Integr Comp Physiol.

[CR17] Guijarro-Munoz I, Compte M, Alvarez-Cienfuegos A, Alvarez-Vallina L, Sanz L (2014). Lipopolysaccharide activates Toll-like receptor 4 (TLR4)-mediated NF-kappaB signaling pathway and proinflammatory response in human pericytes. J Biol Chem.

[CR18] Long TM, Nisa S, Donnenberg MS, Hassel BA (2014). Enteropathogenic Escherichia coli inhibits type interferon- and RNase L-mediated host defense to disrupt intestinal epithelial cell barrier function. Infect Immun.

[CR19] Malago JJ, Koninkx JFJG, van Dijk JE (2002). The heat shock response and cytoprotection of the intestinal epithelium. Cell Stress Chaperones.

[CR20] Mariathasan S, Weiss DS, Dixit VM, Monack DM (2005). Innate immunity against Francisella tularensis is dependent on the ASC/caspase-1 axis. J Exp Med.

[CR21] Martinon F, Tschopp J (2004). Inflammatory caspases: linking an intracellular innate immune system to auto-inflammatory diseases. Cell.

[CR22] Mestecky J (1987). The common mucosal immune system and current strategies for induction of immune responses in external secretions. J Clin Immunol.

[CR23] Nemeth E, Fajdiga S, Malago J, Koninkx J, Tooten P, van Dijk J (2006). Inhibition of Salmonella-induced IL-8 synthesis and expression of Hsp70 in enterocyte-like Caco-2 cells after exposure to non-starter lactobacilli. Int J Food Microbiol.

[CR24] Newton K, Dixit VM (2012). Signaling in innate immunity and inflammation. CSH Perspect Biol.

[CR25] Pearce SC, Mani V, Boddicker RL, Johnson JS, Weber TE, Ross JW, Rhoads RP, Baumgard LH, Gabler NK (2013). Heat stress reduces intestinal barrier integrity and favors intestinal glucose transport in growing pigs. PLoS One.

[CR26] Perrin AJ, Jiang X, Birmingham CL, So NS, Brumell JH (2004). Recognition of bacteria in the cytosol of mammalian cells by the ubiquitin system. Curr Biol.

[CR27] Quinteiro-Filho WM, Gomes AV, Pinheiro ML, Ribeiro A, Ferraz-de-Paula V, Astolfi-Ferreira CS, Ferreira AJ, Palermo-Neto J (2012). Heat stress impairs performance and induces intestinal inflammation in broiler chickens infected with Salmonella Enteritidis. Avian Pathol.

[CR28] Quinteiro-Filho WM, Calefi AS, Cruz DSG, Aloia TPA, Zager A, Astolfi-Ferreira CS, Piantino Ferreia JA, Sharif S, Palermo-Neto J (2017). Heat stress decreases expression of the cytokines, avian alfa-defensins 4 and 6 and Toll-like receptor 2 in broiler chickens infected with Salmonella Enteritidis. Vet Immunol Immunopathol.

[CR29] Ramachandran G (2014). Gram-positive and gram-negative bacterial toxins in sepsis. Virulence.

[CR30] Rychlik I, Barrow PA (2005). Salmonella stress management and its relevance to behaviour during intestinal colonisation and infection. FEMS Microbiol Rev.

[CR31] Sansonetti PJ, Phalipon A, Arondel J, Thirumalai K, Banerjee S, Akira S, Takeda K, Zychlinsky A (2000). Caspase-1 activation of IL-1beta and IL-18 are essential for Shigella flexneri-induced inflammation. Immunity.

[CR32] Sellin ME, Muller AA, Felmy B, Dolowschiak T, Diard M, Tardivel A, Maslowski KM, Hardt WD (2014). Epithelium-intrinsic NAIP/NLRC4 inflammasome drives infected enterocyte expulsion to restrict Salmonella replication in the intestinal mucosa. Cell Host Microbe.

[CR33] Tojima Y, Fujimoto A, Delhase M, Chen Y, Hatakeyama S, Nakayama K, Kaneko Y, Nimura Y, Motoyama N, Ikeda K, Karin M, Nakanishi M (2000). NAK is an IkappaB kinase-activating kinase. Nature.

[CR34] Varasteh S, Braber S, Akbari P, Garssen J, Fink-Gremmels J (2015). Differences in susceptibility to heat stress along the chicken intestine and the protective effects of galacto-oligosaccharides. PloSOne.

[CR35] Wittebole X, Castanares-Zapatero D, Laterre PF (2010). Toll-like receptor 4 modulation as a strategy to treat sepsis. Mediat Inflamm.

[CR36] Wotzka SY, Nguyen BD, Hardt WD (2017). *Salmonella typhimurium* diarrhea reveals basic principles of enteropathogen infection and disease-promoted DNA exchange. Cell Host Microbe.

[CR37] Yoo C, Lee S, Lee C, Kim YM, Han SK, Shim Y (2000). Anti-inflammatory effect of heat shock protein induction is related to stabilization of IkBa through preventing IkB kinase activation in respiratory epithelial cells. J Immunol.

